# Clinical performance of Hedia Diabetes Assistant bolus calculator for diabetes management: a real-world retrospective cohort study

**DOI:** 10.3389/fdgth.2025.1430744

**Published:** 2025-03-27

**Authors:** Kenney Fehrenkamp Pedersen, Anne Østerskov, Sabrina Mai Nielsen, Gkikas Karagkounis, Astrid Karnøe Knudsen

**Affiliations:** ^1^ Clinical and Medical Affairs, Hedia ApS, Copenhagen, Denmark; ^2^ Section for Biostatistics and Evidence-Based Research, The Parker Institute, Bispebjerg and Frederiksberg Hospital, Copenhagen, Denmark

**Keywords:** bolus calculator, type 1 diabetes mellitus, digital technologies, glycemic control, hypoglycemia

## Abstract

**Introduction:**

Individuals living with type 1 diabetes are at risk of long-term complications related to chronic hyperglycemia. Tight glycemic control is recommended but can increase the risk of iatrogenic hypoglycemia. Hedia Diabetes Assistant (HDA) is a bolus calculator that provides users with bolus insulin recommendations based on personalized settings. We aimed to investigate the effects of HDA on a known risk index of hypoglycemia.

**Methods:**

New users from 2019 to 2021 were included if they fulfilled the following criteria: age ≥18 years, ≥5 logs/1st week of use, and ≥1 log for glucose, carbohydrate, and insulin. User data was extracted from the HDA database. The prespecified primary endpoint was change in the Low Blood Glucose Index (LBGI) after 12 weeks of use. Secondary endpoints were changes in the High Blood Glucose Index (HBGI) and eA1c. An exploratory endpoint was to maintain potential improvements in LBGI after 25 weeks. A repeated-measures mixed model with a log-transformation was used.

**Results:**

A total of 1,342 users were included. The mean age was 43.4 years (SD 14.7) with 52.3% being female. After 12 weeks, LBGI significantly improved from 0.73 to 0.61 (17% decrease, *P* < 0.001) with no significant changes in HBGI, and eA1c. From week 12 to 25, LBGI decreased from 0.61 to 0.55 (10%, *P* = 0.107).

**Conclusions:**

Users of HDA experienced statistically significant improvement in LBGI after 12 weeks with no changes in HBGI and eA1c, which was successfully maintained after 25 weeks. These results suggest a decreased risk of hypoglycemia when using HDA.

## Introduction

Tight glycemic control is recommended for individuals with type 1 diabetes but can increase the risk of iatrogenic hypoglycemia (1). Insulin bolus calculators can aid in this, by positively impacting both glycemic control and the risk of hypoglycemia (2). Multiple insulin bolus calculator apps exist and most have similar functions. They typically rely on advanced carbohydrate counting for carbohydrate intake and blood glucose measurements to correct glucose excursions and can also take insulin on board into account (3).

This study aimed to examine the clinical performance of the insulin bolus calculator, Hedia Diabetes Assistant (HDA), on glycemic control in adults with type 1 diabetes.

## Methods

This was a real-world retrospective cohort study conducted on new users of HDA with type 1 diabetes. The users were included if they started using HDA between 1st of January 2019 and 31st of December 2021, were not <18 years of age, had opted to share their data for research purposes, had made at least five logs during the first week of use and had logged blood glucose, carbohydrates, and insulin at least once. Data were collected from the HDA database, which contains prospectively collected data on user interactions with HDA (blood glucose measurements, carbohydrate ingestion, physical activity, and insulin recommendations).

### Hedia diabetes assistant

HDA is a CE-marked Class IIb medical device that calculates insulin bolus doses using blood glucose, carbohydrates, and physical activity based on personalized settings. Blood glucose is manually entered or synced from a supported blood glucose meter, while carbohydrates and physical activity are entered manually. HDA also has a food database to assist users with calculating the amount of carbohydrates.

HDA calculates a meal bolus based on carbohydrates and the insulin-to-carbohydrate ratio, and a correction bolus based on blood glucose measurements and the insulin sensitivity factor. Insulin on board is subtracted from the correction bolus. Physical activity reduces the final bolus recommendation based on duration and intensity (4). HDA will recommend carbohydrates instead of insulin in case of hypoglycemia.

### Endpoints

The primary endpoint was the risk of hypoglycemia assessed by the Low Blood Glucose Index (LBGI) after 12 weeks. The secondary endpoints were the risk of hyperglycemia assessed by the High Blood Glucose Index (HBGI) and glycemic control assessed by estimated A1c (eA1c, calculated based on ≥10 logged blood glucose values per week) after 12 weeks.

Exploratory endpoints included analyses of changes from weeks 12 to 25 in the abovementioned endpoints and the distribution of LBGI and HBGI based on risk categories at weeks 0, 12, and 25. Users with suboptimal glycemic control [users with eA1c ≥64 mmol/mol (≥8%) at baseline] were further analyzed as a subgroup.

The LBGI and HBGI are established metrics used to quantify the risk of hypo- and hyperglycemia by using sparse self-monitoring blood glucose data. They are calculated using logarithmic transformation of the blood glucose scale, which broadens the hypoglycemic range and shortens the hyperglycemic range, thereby symmetrizing the blood glucose scale. The LBGI has been shown to predict future episodes of hypoglycemia and can be categorized as low (<2.5), moderate (2–5 - 5), and high risk (>5). The HBGI is correlated with A1c and can also be categorized as low (<4.5), moderate (4.5–9), and high risk (>9) (5).

### Statistical methods

The sample size was based on the number of eligible subjects in the HDA database.

Baseline characteristics were summarized as means with standard deviations (SD) when normally distributed or as medians with interquartile ranges in case of skewed data. Normality was assessed using Q-Q plots, and histograms.

The outcomes were analyzed using repeated-measures mixed models, which indirectly handle missing data. Since the distributions of the primary and secondary outcomes were positively skewed, a log transformation was applied before the analysis (6). The use of log transformation helps address the skewness of the data, allowing for a more appropriate analysis using the mixed model. To incorporate zero values, one was added to all scores before transformation. The means and contrasts were back-transformed (exp[Y]-1), to obtain the geometric means and geometric mean ratios (GMR) which summarize the relative changes within the group over time. All statistical tests were two-sided and were performed using a 5% statistical significance level. Instead of adjusting for multiple tests, a serial gatekeeping procedure was employed.

All analyses were performed using the R statistical software (R Foundation) version 4.0.3.

## Results

**Figure 1:**
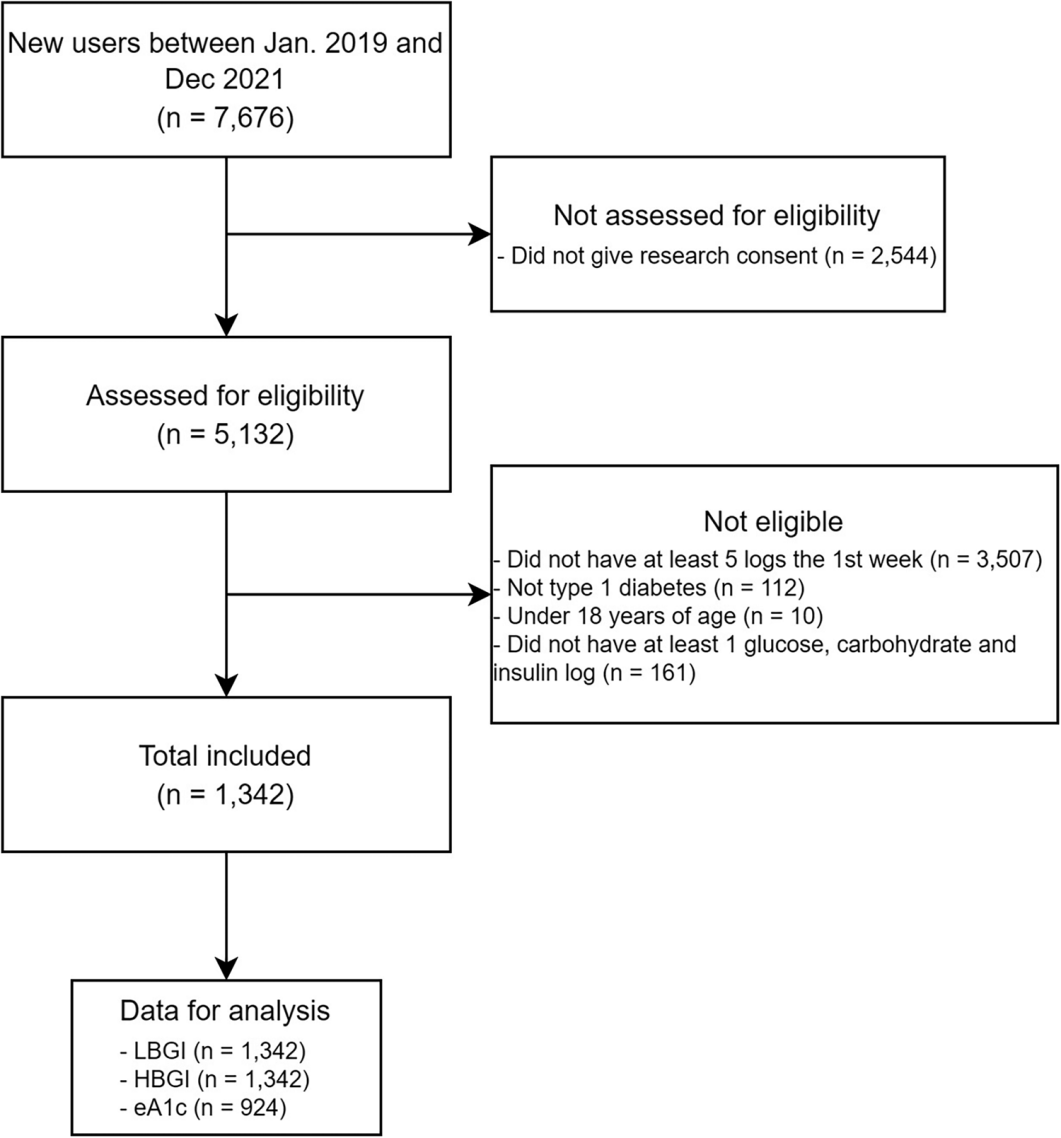
Flow-chart of user inclusion. eA1c, estimated A1c; HBGI, high blood glucose index; LBGI, low blood glucose index.

In total, there were 7,676 new users between the 1st of January 2019 and the 31st of December 2021 (Figure 1). A total of 1,342 eligible users were included in this study. Baseline characteristics are summarized in Table 1.

**Table 1:** Population characteristics.

Characteristics	*N*	Study population
Age (years), mean (SD)	607	43.4 (14.7)
Women, *n* (%)	1,342	702 (52.3%)
Body weight (kg)	620	76.0 (66.0–90.0)
BMI (kg/m^2^)	589	25.4 (22.7–29.1)
Insulin sensitivity factor (mmol/L/unit)	1,318	2.1 (1.7–3.0)
Insulin-to-carb-ratio (g/unit)	1,317	10.0 (7.5–13.0)
Type of rapid-acting-insulin	1,310	
Apidra, *n* (%)		21 (1.6%)
Fiasp, *n* (%)		183 (14.0%)
Humalog, *n* (%)		72 (5.5%)
Novorapid, *n* (%)		1,020 (77.9%)
Other, *n* (%)		14 (1.1%)

Data on population characteristics are median (IQR) unless otherwise stated.

BMI, body mass index; SD, standard deviation.

### Primary and secondary outcomes

Data for LBGI and HBGI were available for all users at baseline but availability decreased to 549 users (40.9%) at week 12 and 458 (34.1%) at week 25. Data for eA1c were available for 924 (68.9%) users at baseline, and the availability decreased to 454 (33.8%) and 391 (29.1%) at week 12 and 25, respectively.

**Figure 2:**
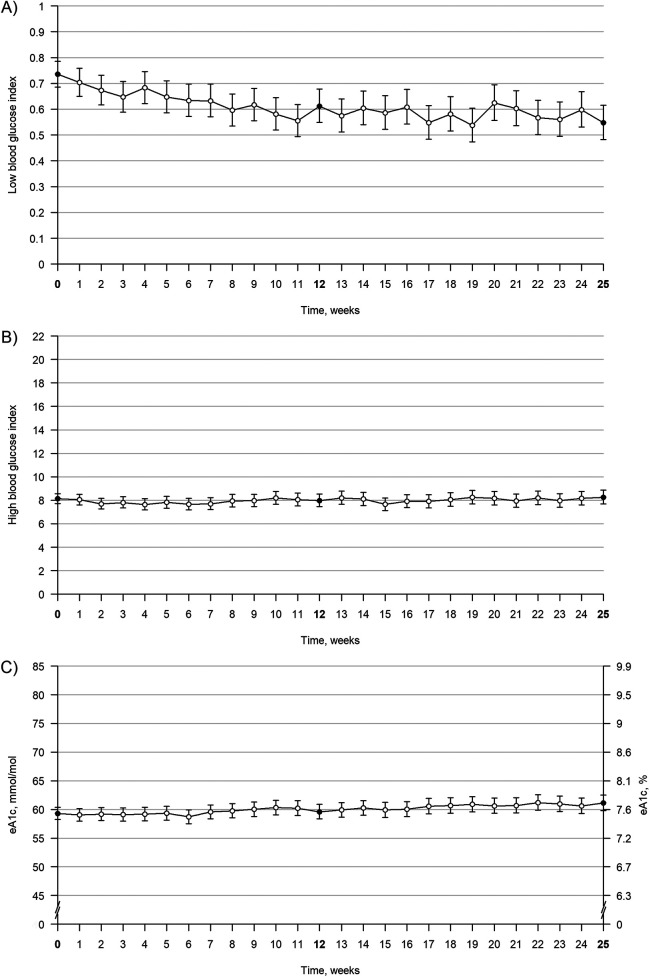
Trajectories of LBGI, eA1c, and HBGI from week 0 to week 25. Trajectories of LBGI, eA1c, and HBGI from week 0 to week 25. The figure shows values over time for LBGI **(A)**, HBGI **(B)**, and eA1c **(C)** Bold week numbers and black points indicate time points of interest, and error bars indicate 95% confidence intervals. The values are geometric means. eA1c, estimated A1c; HBGI, high blood glucose index; LBGI, low blood glucose index.

From week 0 to 12, LBGI significantly decreased by 17% (GMR: 0.83; *P* < 0.001). No statistically significant changes were found for HBGI (GMR: 0.98; *P* = 0.547) or eA1c (GMR: 1.01; *P* = 0.594) (Figure 2 and Table 2).

**Table 2:** Clinical performance after 12 weeks of using the hedia diabetes assistant.

Outcomes	*N*	Week 0^a^	Week 12	Contrast	*P*-value
LBGI	1,342	0.73 (0.69–0.79)	0.61 (0.55–0.68)	GMR, 0.83	<0.001
HBGI	1,342	8.14 (7.72–8.57)	7.98 (7.46–8.54)	GMR, 0.98	0.547
eA1c, mmol/mol	1,069	59.3 (58.3–60.4)	59.6 (58.4–60.9)	GMR, 1.01	0.594

Data are geometric mean (95%CI) and geometric mean ratios for the contrast between the two time points.

BMI, body mass index; SD, standard deviation, eA1c, estimated A1c; GMR, geometric mean ratio; HBGI, high blood glucose index; LBGI, low blood glucose index.

^a^ Week 0 covers the first week of using the Hedia Diabetes Assistant.

### Exploratory endpoints

From week 12 to 25, no changes were found in LBGI or HBGI. However, eA1c increased slightly (GMR: 1.03) during this period.

At week 0, a total of 3.6% of users were at high risk of hypoglycemia and 9.1% were at moderate risk. At week 12, this decreased to 1.4% at high risk and 8.2% at moderate risk. At week 25, this had further decreased to 1% at high risk and 6.3% at moderate risk. A similar trend was not observed for HBGI, where a total of 49.9% were at high risk of hyperglycemia at week 0, which slightly increased to 51.1% and 56.3% at week 12 and 25, respectively (Supplementary Material).

### Subgroup analysis

In total, 335 (25%) users had suboptimal glycemic control at baseline. From week 0 to 12, LBGI increased by 31% (GMR: 1.31, *P* < 0.05), while HBGI and eA1c decreased by 32% (GMR: 1.68, *P* < 0.001) and 15% [GMR: 0.85; 11.8 mmol/L (1.1%-points), *P* < 0.001], respectively.

## Discussion

The main finding of this study was a statistically significant 17% decrease in LBGI at week 12, indicating that users experienced fewer episodes of hypoglycemia and/or less severe episodes of hypoglycemia. An improvement in LBGI suggests that users are at less risk of future episodes of hypoglycemia (7). Users were able to achieve this improvement without increasing their HBG or eA1c after 12 weeks, however, eA1c was slightly increased after 25 weeks. Evaluating the clinical implications of this is difficult, as the users generally had a low risk of hypoglycemia at baseline. However, the potential clinical relevance is supported by the decrease in users at moderate and high risk of hypoglycemia. The small increase in eA1c from weeks 12 to 25 is unlikely to be clinically significant (8).

These findings align with a recent systematic review and meta-analysis that found insulin bolus calculators (not limited to smartphone apps) to lead to small improvements in A1c and LBGI (2). However, the improvement in A1c in this study was only seen in the subgroup with suboptimal glycemic control. They experienced an 11.8 mmol/L (1.1%-points) reduction in A1c, although at the cost of increased LBGI, suggesting an elevated risk of hypoglycemia for these users. While large cohort studies have highlighted the clinical benefit of incremental improvements in A1c - demonstrating that every 10 mmol/mol (1%) increase in A1c is associated with a 30% increased risk of mortality from ischemic heart disease (9) - the accompanying rise in risk of hypoglycemia underscores the challenge of optimising glycemic control while minimising hypoglycemia.

This study had a relatively low retention rate, which is a common challenge for mobile health apps (10). The inclusion criteria of at least five logs during the first week were designed to exclude users who downloaded HDA merely to test it. However, this approach likely did not fully eliminate the inclusion of users testing HDA, which could contribute to the observed attrition. This may reduce the generalizability of the findings, since retained users could differ from the total population in meaningful ways. Additionally, the study only included users of HDA and lacked a control group and data on how users managed their diabetes before starting HDA. The lack of a control group limits the ability to distinguish the specific impact of HDA on clinical outcomes from other confounding factors.

While the repeated-measures mixed model accounted for individual variability and the correlation of repeated measures, it did not control for potential confounding factors such as diet, lifestyle changes, or existing diabetes management, as these data were not available. This limits the generalizability of the results to a broader population. Furthermore, the lack of data on confounding variables means that changes observed in glycemic outcomes could be partly attributable to unmeasured factors, making it difficult to attribute outcomes solely to the app's use.

This study relied on user-reported data. While this might introduce inaccuracies, particularly due to the subjective nature of carbohydrate counting, it is important to highlight that the user-reported data served as input to the users' insulin calculations. As such, even if the dataset is limited to the entries users chose to input, this reflects real-world diabetes management and provides a realistic assessment of the app's impact in real-world conditions.

In summary, despite the limitations, these results suggest that HDA has potential clinical benefits by lowering the risk of hypoglycemia and also improving glycemic control for users with suboptimal glycemic control. Future prospective studies may investigate the effects in subgroups with a high risk of hypoglycemia, as these were not well represented in this study, as well as users with suboptimal glycemic control, as they appear to potentially have the most benefits in terms of glycemic control. Future studies should also aim to collect more comprehensive data on potential confounding factors to better control for these variables.

## Data Availability

The raw data supporting the conclusions of this article will be made available by the authors upon reasonable request.
