# Supplementary material for: Clinical performance of Hedia Diabetes Assistant bolus calculator for diabetes management: a real-world retrospective cohort study
Source: Front Digit Health. 2025 Mar 27;7:1430744. doi: 10.3389/fdgth.2025.1430744 (PMC11983442; doi:10.3389/fdgth.2025.1430744)
Supplement: Supplementary file 1 [file Table1.docx]

**Supplemental materials**

Table S1. Risk matrix showing the distribution of users in risk categories (risk of hypo- and hyperglycemia) based on LBGI and HBGI values.

| Week 0 | | HBGI | | |
| --- | --- | --- | --- | --- |
|  |  | Low (<4.5) | Moderate (4.5-9.0) | High (>9.0) |
| LBGI | Low (<4.5) | 240 (17.9) | 306 (22.8) | 625 (46.6) |
|  | Moderate (4.5-9.0) | 62 (4.6) | 27 (2.0) | 33 (2.5) |
|  | High (>9.0) | 26 (1.9) | 12 (0.9) | 11 (0.8) |
| Week 12 | | HBGI | | |
|  |  | Low (<4.5) | Moderate (4.5-9.0) | High (>9.0) |
| LBGI | Low (<4.5) | 108 (19.7) | 120 (21.9) | 268 (48.8) |
|  | Moderate (4.5-9.0) | 22 (4.0) | 13 (2.4) | 10 (1.8) |
|  | High (>9.0) | 4 (0.7) | 1 (0.2) | 3 (0.5) |
| Week 25 | | HBGI | | |
|  |  | Low (<4.5) | Moderate (4.5-9.0) | High (>9.0) |
| LBGI | Low (<4.5) | 85 (18.6) | 95 (20.7) | 244 (53.3) |
|  | Moderate (4.5-9.0) | 7 (1.5) | 10 (2.2) | 12 (2.6) |
|  | High (>9.0) | 2 (0.4) | 1 (0.2) | 2 (0.4) |

Values are n (%).

HBGI, high blood glucose index; LBGI, low blood glucose index.
